# Ultra-widefield retinal imaging for early detection of asymptomatic cytomegalovirus retinitis after allogeneic hematopoietic stem cell transplantation: a prospective pilot feasibility study

**DOI:** 10.1186/s12348-026-00585-y

**Published:** 2026-04-28

**Authors:** Aina Moll-Udina, Alexandra Pedraza, Lucia Miguel-Escuder, Maria Suárez-Lledó, Javier Nogués-Castell, Francesc Fernández Avilés, María Borrell Balagué, Mireia Hereu de Batlle, Montserrat Morató Vilaseca, Juan Pablo Figueroa-Vercellino, Víctor Llorenç, Alfredo Adán

**Affiliations:** 1 Institut Clínic d’Oftalmologia. Hospital Clínic de Barcelona, Barcelona, Spain; 2 Institut d’Investigacions Biomèdiques August Pi i Sunyer (IDIBAPS), Fundació Clínic per a la Recerca Biomèdica (FCRB), Universitat de Barcelona, Barcelona, Spain; 3 Department of Hematology, Hospital Clínic of Barcelona, Barcelona, Spain; 4 Obstetricia i Neonatologia (ICGON), Institut Clínic de Ginecologia, Hospital Clínic de Barcelona, Barcelona, Spain; 5 Ocular Inflammation and Infection Section, Institut Clínic d’Oftalmologia. Hospital Clínic de Barcelona. Barcelona, Sabino Arana 1, Barcelona, 08028 Spain

**Keywords:** Cytomegalovirus infection, Cytomegalovirus retinitis, Cytomegalovirus retinitis screening, Ultra-wide field imaging, Hematopoietic stem cell transplantation

## Abstract

**Background:**

To evaluate the feasibility and potential clinical utility of ultra-widefield (UWF) retinal imaging as a screening strategy for the early detection of asymptomatic cytomegalovirus retinitis (CMVR) in patients with systemic cytomegalovirus (CMV) viremia after allogeneic hematopoietic stem cell transplantation (alloHSCT).

**Methods:**

This prospective, single-center pilot study included adult alloHSCT recipients with documented CMV replication within the previous 12 months and no ocular symptoms. Patients underwent UWF retinal imaging (Optomap^®^) at the time of CMV detection and during follow-up. Demographic, hematologic, immunologic, and virologic data were collected. Retinal images were assessed by a uveitis specialist, and patients with suspected retinal involvement underwent targeted ophthalmologic evaluation and treatment.

**Results:**

Thirty-nine alloHSCT recipients (mean age 48.8 years; 51.3% male) were screened using UWF imaging. CMVR was identified in one asymptomatic patient (2.6%), presenting with peripheral retinal periphlebitis and hemorrhages 251 days after alloHSCT. Notably, CMV PCR in peripheral blood was negative at the time of retinal diagnosis, while aqueous humor PCR confirmed CMV infection. Early detection through UWF imaging enabled prompt systemic antiviral therapy, resulting in complete resolution of retinal lesions and preservation of visual acuity.

**Conclusions:**

In this prospective pilot study, UWF retinal imaging proved to be a feasible, non-invasive approach for screening asymptomatic alloHSCT recipients with prior CMV replication. Although the incidence of CMVR was low, UWF imaging allowed detection of early peripheral retinal involvement that would likely have been missed by symptom-based surveillance or blood PCR monitoring alone. These findings support the potential role of targeted UWF screening in selected high-risk patients and justify further evaluation in larger, multicenter prospective studies.

## Introduction

Cytomegalovirus (CMV) infection remains one of the most frequent and clinically relevant complications following allogeneic hematopoietic stem cell transplantation (alloHSCT). Despite the widespread use of prophylactic and pre-emptive antiviral strategies, CMV reactivation continues to occur in a substantial proportion of recipients and may progress to end-organ disease in susceptible patients [1, 2]. Among the spectrum of CMV-related complications, cytomegalovirus retinitis (CMVR) is uncommon but potentially devastating, as it can lead to irreversible visual loss if diagnosis and treatment are delayed [3–6].

In the era prior to effective antiretroviral therapy, CMVR was predominantly observed in patients with acquired immune deficiency syndrome. However, its epidemiology has shifted, and CMVR is now increasingly reported in non-HIV immunocompromised populations, particularly in alloHSCT recipients and solid organ transplant patients [6–8]. Although the overall incidence of CMVR after alloHSCT is relatively low, ranging from 0.1% to 4% across published series [3], visual outcomes are often poor, especially when diagnosis occurs after posterior pole involvement [9].

Current post-transplant CMV surveillance strategies are primarily based on serial quantitative polymerase chain reaction (PCR) testing in peripheral blood, allowing early detection of viral replication and initiation of pre-emptive therapy [5]. While this approach has significantly reduced the incidence of systemic CMV disease, it does not reliably prevent CMVR. Previous studies have shown that CMV retinitis may develop despite negative systemic CMV markers in peripheral blood [3, 4]. As a result, symptom-driven ophthalmologic referral may fail to identify CMVR at a stage amenable to optimal visual preservation.

The gold standard for CMVR diagnosis remains dilated fundus examination by an experienced ophthalmologist. However, systematic funduscopic screening of asymptomatic alloHSCT recipients is not routinely implemented, and there are no established guidelines defining its indication or periodicity. Ultra-widefield (UWF) retinal imaging has emerged as a non-invasive technique capable of capturing up to 200 degrees of the retina in a single image, enabling improved visualization of peripheral retinal pathology compared with conventional fundus photography [10, 11]. Previous studies have suggested that UWF imaging may facilitate detection and monitoring of peripheral CMVR lesions, but its role as a screening tool in the alloHSCT population has not been systematically explored.

The aim of the present study was therefore to evaluate the feasibility and potential clinical utility of a CMVR screening strategy using UWF retinal imaging in asymptomatic alloHSCT recipients with documented CMV replication. By prospectively applying this imaging modality during the first year after transplantation, we sought to assess its ability to detect early peripheral retinal involvement that might be missed by symptom-based surveillance or blood PCR monitoring alone.

## Methods

### Study design

We performed an observational, prospective, single-center and multidisciplinary study in which the Hematology and Ophthalmology departments of the Hospital Clínic de Barcelona (Spain) participated between October 2019 and January 2023.

This study was done in compliance with the Declaration of Helsinki and its further amendments (October 2023). The study protocol was approved by the Ethics Committee of Clinic Hospital of Barcelona. All study participants provided written informed consent for the use of the medical data for research purpose.

Adult patients aged ≥ 18 years who had undergone alloHSCT within the previous year, with detectable CMV DNA in peripheral blood and no recent-onset ophthalmologic symptoms, were included. Patients were included regardless of CMV viral load level at the time of detection. Antiviral therapy was administered according to institutional CMV management protocols in our hematology department, in accordance with ECIL guidelines and current recommendations. Briefly, CMV viremia was monitored weekly during the first 100 days after transplantation and every two weeks from day 100 to day 180. In patients receiving corticosteroid therapy due to graft-versus-host disease (GVHD), CMV PCR was performed twice weekly. Antiviral treatment was initiated when CMV viral load exceeded 1,000 copies/mL. Patients requiring treatment received valganciclovir 900 mg daily (or foscarnet in case of contraindication) for two weeks or until CMV PCR became negative, according to standard clinical practice.

Ocular exclusion criteria were corneal or lens opacity that precluded the visualization of UWF images. Likewise, patients whose poor general condition did not allow the necessary collaboration to obtain the Optomap^®^ images or to attend follow-up visits were also excluded.

Ophthalmological examination included Snellen best corrected visual acuity (BCVA) and a retinal imaging consisting of an ultrawide field pseudocolor retinography (UWF^®^) and blue autofluorescence, performed with Optomap^®^ after pupillary dilation with tropicamide. Images were stored in Optomap^®^ commercial viewer Optos V^2^ Vantage Pro. Retinal images were analysed by a trained retina specialist. Those patients with retinal lesions were referred to uveitis section for a proper follow-up and treatment. The diagnosis of CMVR was clinical, based on the SUN key criteria for CMV retinitis [12].

Ophthalmologic screening was initiated after the detection of CMV viremia in peripheral blood.

Other ancillary tests (e.g.: PCR in aqueous humor) could be performed if necessary to confirm the diagnosis, without depending on the study. In cases where Optomap^®^ UWF image showed retinitis lesions compatible with CMVR, the dose of oral therapy was increased to 900 mg twice a day was extended for more than two weeks or switched to intravenous ganciclovir (5 mg/kg every 12 h). Patients who did not present lesions on the Optomap^®^ were followed up with a UWF^®^ retinography every 3 months for a year (five Optomap^®^ images throughout the study).

### Data collection

Patient demographic and clinical data were collected prospectively and confidentially: age, sex, ethnicity, hematologic disease, donor type, graft-versus-host disease (GVHD) prophylaxis, serological risk (due to CMV compatibility between receptor and donor), presence of chronic or acute GVHD, corticoids therapy, CD8 and CD4 count, ophthalmologic antecedents, presence of CMV disease (except retinitis), number of previous CMV reactivations, number of days from the transplant, number of CMV copies/mL in blood, treatment and days until CMV-PCR became negative. Number of retinal lesions and duration.

### Data analysis

A descriptive analysis was done using appropriate parameters for each variable. To describe the qualitative variables, absolute frequencies, and percentages were used. The description of quantitative variables was performed using the mean and standard deviation (SD). Given the exploratory and feasibility-focused nature of this pilot study, analyses were limited to descriptive statistics, and no formal hypothesis testing was performed.

## Results

### Study population

Thirty-nine patients with CMV replication in blood after alloHSCT between October 2019 and January 2023 were included. The mean age was 48.8 years (23–70, SD 13.9), 20 (51.3%) were men. Baseline demographics are described in Table 1.

**Table 1:** Baseline demographic and transplant-related characteristics of the study population

Baseline Demographics	*n* (%)
Age: mean (SD)	48.8 (13.9)
Sex: male / female	20 /19
Origin	
Caucasian	35 (89.7)
Hispanic	3 (7.7)
African	1 (2.6)
Donor type	
Blood related	12 (30.8)
Not Blood related	23 (58.9)
Haploidentical	4 (10.3)
Serological Risk	
Low (D-/R-)	18 (46.2)
Medium (D+/R + or D+/R-)	21 (53.8)
High (D-/R+)	0
Hematological Disease	
Acute myeloblastic leukemia	10 (25.6)
Acute lymphoblastic leukemia	10 (25.6)
Chronic myeloblastic leukemia	1 (2.6)
Plasma cell leukemia	1 (2.6)
Hodgkin lymphoma	2 (5.1)
Non-Hodgkin lymphoma	3 (7.7)
Myelomonocytic chronic leukemia	4 (10.3)
Myelofibrosis after TE	1 (2.6)
Myelodysplastic syndrome	7 (17.9)

D: Donor; R: Receptor; -: CMV negative; +: CMV positive; TE: essential thrombocythemia.

Sixteen (41%) Patients had chronic GVHD. Seven of them presented cutaneous involvement, four had intestinal involvement, two patients had gastric involvement, two had both cutaneous and intestinal and one patient had both cutaneous and gastric. Three patients (7.7%) had acute GVHD, one of them with conjunctival involvement. Eighteen (46.1%) patients received oral corticoids either for acute or chronic GVHD. At the time of first CMV detection in blood, all patients were receiving prophylaxis against GVHD, either with post-trasplant cyclophosphamide (PTCy) plus tacrolimus after transplantation, with or without mycophenolate mofetil, or with cyclosporine and methotrexate. So at the time of the CMV infection all patients were receiving immunosupresion with tacrolimus or mycophenolate.

At the time of CMV viremia, the mean CMV load was 1889.2 ± 444 copies/mL. Seven patients had CMV viral loads between 500 and 1000 copies/mL, while twenty-one patients had viral loads exceeding 1000 copies/mL. Eleven patients had viral loads below 500 copies/mL. Mean CD8 + lymphocyte count was 923,6cells/mm^3^ and mean CD4 + count was 192.1 cells/mm^3^. Other variables are detailed in Table 2.

**Table 2:** Virological, immunological, and clinical parameters at the time of CMV viremia

	Mean	Standard Deviation	Range
CMV copies (copies/ml)	1889,2	444	77–12,300
CD8+ (cells/mm^3^)	923.6	748.1	50–2638
CD4+ (cells/mm^3^)	192.1	242.6	2–1217
Time between HSCT and CMV blood replication (days)	30.9	35.8	2–216
Time to CMV PCR negativization (days)	184	316	24-1505

Three patients also presented extraocular cytomegalic disease, all of them due to gastrointestinal involvement. Thirty-four patients received treatment for the CMV infection, the most common treatment was valganciclovir used either in monotherapy more frequently or combined with other antivirals. More details about the treatment are summarized in Table 3. The mean treatment duration was 24 days, with a maximum of 12 week. The mean time to CMV PCR negativization was 184 days. Five patients did not receive antiviral treatment because CMV replication remained at low levels (< 1000 copies/mL) and did not meet the institutional treatment threshold.

**Table 3:** Antiviral treatment regimens and outcomes in alloHSCT recipients with CMV replication

Treatment received	*n* (%)
No treatment	5 (12.8%)
Valganciclovir	16 (41%)
Valganciclovir + Foscarnet	10 (25,6%)
Maribavir	3 (7.7%)
Cidofovir	1 (2.6%)
Foscarnet	1 (2.6%)
Ganciclovir + Foscarnet	3 (7.7%)

The mean time between HSCT and the first UWF^®^ retinography was 91.8 days (47–294 +/- 57.6). Eleven patients (28.2%) completed all five scheduled UWF^®^ follow-up examinations performed every three months. Four Optomap^®^ were made in nine patients (23.1%), three in 5 patients (12.8%), two in 5 patients, and one Optomap^®^ in 9 patients. Four patients died during the year of the study. The other patients who could not complete the study (five Optomap^®^) were lost to follow-up, mainly due to worsening of their general condition. Patients underwent scheduled ophthalmologic follow-up with ultra-widefield retinal imaging every three months during the first year after first CMV replication detection.

**Fig. 1:**
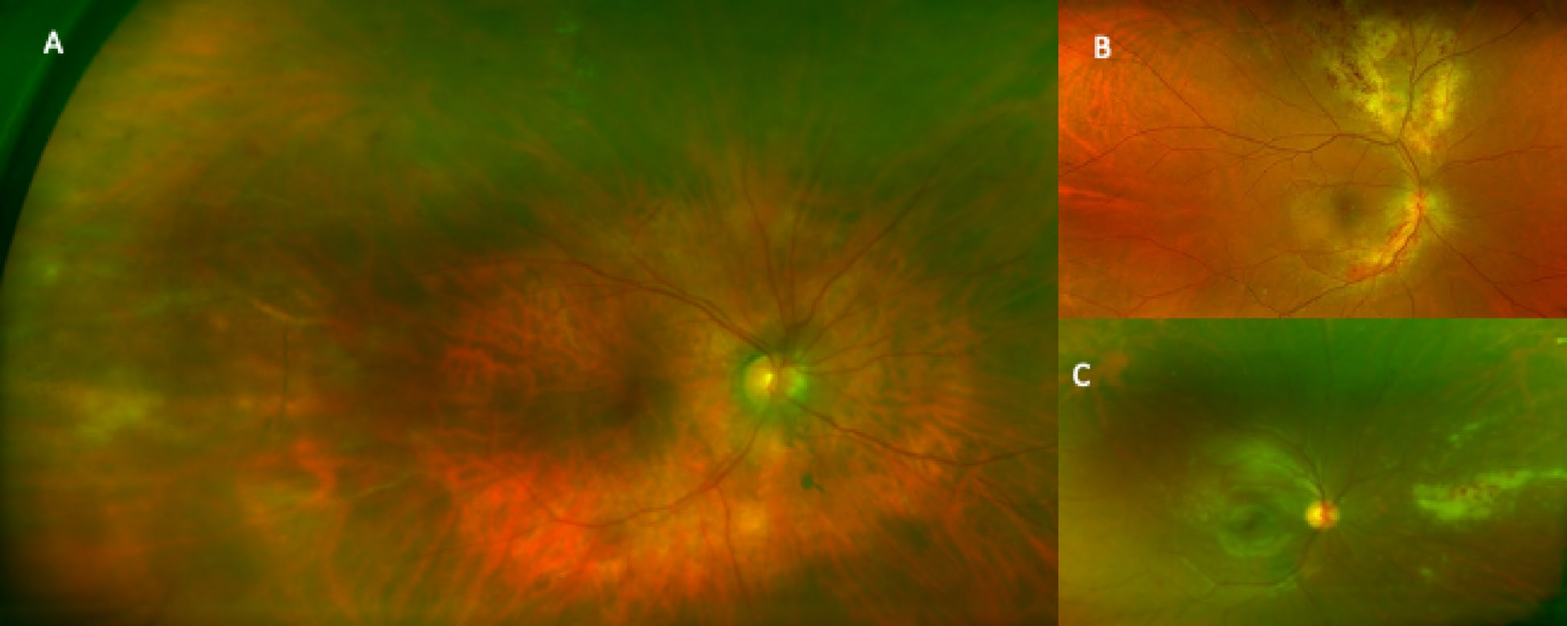
UWF retinal images illustrating different clinical patterns of cytomegalovirus retinitis (CMVR). (**A**) Periphlebitis pattern: peripheral perivascular inflammation with associated intraretinal hemorrhages, corresponding to the asymptomatic case described in the present study. (**B**) Fulminant pattern: extensive areas of retinal necrosis with prominent flame-shaped hemorrhages along the vascular arcades, showing the characteristic “brush-fire” appearance. (**C**) Granular pattern: scattered small white retinal opacifications in the peripheral retina with minimal associated hemorrhage

CMV retinitis was detected in one patient (2.6%) during the study period (Fig. 1). A CMV positive viremia was detected 48 days after the transplantation (2810copies/mL). He received treatment with valganciclovir and foscarnet for four weeks at that time. In the first two UWF^®^ retinographies, non-specific punctate hemorrhages in the peripheral retina were observed in both eyes, with no signs of retinitis or vasculitis. At the time of the third capture (251 days post-transplant) an area of periphlebitis and hemorrhages was shown in the periphery of nasal retina in the right eye. Although CMV reactivation had occurred six weeks after transplantation, the patient was not receiving systemic immunosuppressive therapy at the time of CMV viremia or retinal hemorrhage detection, as tacrolimus had been discontinued twelve weeks earlier due to acute renal failure.

BCVA was 20/20 in both eyes at the time of CMVR diagnosis and the patient was asymptomatic. An aqueous humor tap PCR confirmed intraocular CMV replication (14.994 copies/mL). Interestingly, CMV was not detected in blood samples taken at the same time. Oral valganciclovir (900 mg every 12 h) was given for 4 weeks with resolution of the retinal lesions. No intravitreal treatment was required. The patient did not develop cytomegalic disease in other organs.

## Discussion

In this prospective pilot study, UWF^®^ retinal imaging was used to screen allogeneic hematopoietic stem cell transplantation (alloHSCT) recipients with documented CMV blood replication, identifying subclinical cytomegalovirus retinitis (CMVR) in one patient (2.6%). Although asymptomatic CMVR appears to be uncommon, delayed diagnosis has been associated with significant visual complications in previous studies [9, 13].

Interestingly, CMVR in our cohort was detected in the absence of detectable CMV viremia. This observation is consistent with previous reports showing that CMV retinitis may develop despite negative systemic CMV markers [3, 4]. These findings suggest that ocular disease may occur even after apparent systemic viral control and highlight a potential role for ophthalmologic screening strategies beyond blood CMV monitoring alone. However, the optimal duration and target population for post-transplant ophthalmologic screening remain to be determined. This observation raises the question of whether ophthalmologic screening strategies should rely solely on CMV viremia or whether selected high-risk alloHSCT recipients might benefit from ophthalmologic evaluation even after systemic viral replication has resolved.

The interval between alloHSCT and CMVR diagnosis was 251 days, supporting previous reports describing CMVR as a late-onset complication after transplantation. Similar timeframes have been reported by Crippa et al. and Yang et al., while other series have described earlier onset [14, 15]. Variability in reported timing likely reflects differences in patient characteristics, intensity of immunosuppression, immune reconstitution, and surveillance strategies. Importantly, delayed diagnosis of CMVR has been associated with poorer visual outcomes, emphasizing the importance of early detection before posterior pole involvement [3, 9]. A particularly relevant observation in this study was the diagnosis of aqueous high viral load CMVR in an asymptomatic patient with negative CMV PCR in peripheral blood at the time of retinal involvement. This finding highlights the limitations of blood based CMV surveillance strategies for detecting ocular disease and suggests that CMVR may develop despite apparent systemic viral control [16]. In line with our findings, Kim et al. reported that 14.3% of alloHSCT recipients diagnosed with CMVR had negative CMV PCR in peripheral blood at the time of retinitis diagnosis, further supporting the concept that ocular CMV disease may occur in the absence of detectable systemic viremia [3]. Reliance solely on symptom-driven ophthalmologic referral or systemic CMV monitoring may delay the diagnosis of CMVR until more advanced stages of retinal involvement. Previous studies have shown that delayed diagnosis and central retinal involvement are associated with poorer visual outcomes in CMVR [3, 17]. Importantly, any alloHSCT recipient presenting with new ocular symptoms should be promptly referred for ophthalmologic evaluation, regardless of CMV viral load in peripheral blood, as CMVR may occur even in the absence of detectable viremia.

Ultra-widefield retinal imaging provides a wide view of the peripheral retina and may facilitate the detection of early or peripheral CMVR lesions that could be difficult to identify during routine examination. Previous studies have shown that UWF imaging can capture up to 200° of the retina and may be useful for the diagnosis and follow-up of CMV retinitis by allowing better visualization of peripheral lesions [18, 19]. This may be particularly relevant in screening settings, where peripheral and asymptomatic CMVR lesions may otherwise remain undetected. However, UWF imaging should be considered a complementary tool rather than a replacement for conventional dilated fundus examination.

CMVR lesions typically originate in the peripheral retina and may remain asymptomatic until advanced stages, particularly in indolent forms occurring in patients who are not severely immunocompromised [18–20]. In our patient, early peripheral retinal changes were initially subtle and nonspecific, later evolving into periphlebitis compatible with CMVR. UWF^®^ imaging enabled visualization of these peripheral lesions, which might have been overlooked with conventional fundus examination or imaging techniques. These findings support further evaluation of targeted UWF^®^ screening strategies for the detection and monitoring of peripheral CMVR lesions [10, 11].

Given the low incidence of CMVR observed in this cohort, our results do not support universal ophthalmologic screening of all alloHSCT recipients with blood CMV replication. Instead, they suggest that targeted screening strategies focusing on selected high-risk patients may be more appropriate. Established risk factors for CMV reactivation and disease include graft-versus-host disease, prolonged or high-dose corticosteroid therapy, delayed immune reconstitution, and recurrent CMV reactivations [8, 20]. Incorporating these factors into risk-adapted screening protocols may increase the diagnostic yield and clinical relevance of ophthalmologic surveillance.

Several limitations of this study should be acknowledged. The relatively small sample size and the low number of CMVR cases preclude conclusions regarding CMVR incidence or the cost-effectiveness of screening strategies. In addition, incomplete ophthalmologic follow-up in some patients, mainly due to clinical deterioration or death, reflects the frailty of the alloHSCT population [15]. The temporal overlap with the COVID-19 pandemic may have further contributed to loss to follow-up. Finally, as a single-center pilot study, the generalizability of these findings is limited. In addition, patients able to complete repeated ophthalmologic examinations may represent a clinically more stable subgroup of alloHSCT recipients, which could introduce potential selection bias.

In conclusion, this prospective pilot study suggested that UWF^®^ retinal imaging is a feasible and non-invasive tool capable of detecting early, peripheral, and asymptomatic CMVR in selected alloHSCT recipients, including cases with negative CMV PCR in peripheral blood. These findings support further evaluation of targeted UWF^®^ screening strategies in larger, multicenter studies to better define their role within post-transplant surveillance programs.

## Data Availability

The datasets generated and/or analyzed during the current study are included in this published article. Ultra-widefield retinal images are stored at the Institut Clínic d’Oftalmologia (Hospital Clínic de Barcelona) and are available from the corresponding author upon reasonable request.
